# The Positive Writing on Mood States: Empirical Study

**DOI:** 10.1192/j.eurpsy.2025.539

**Published:** 2025-08-26

**Authors:** I. S. Lancia, G. M. Festa, A. Attouchi, F. De Pasquale, M. Manganozzi

**Affiliations:** 1 Interdisciplinary Institute of Advanced Clinical Training «IACT»; 2 Pontifical Faculty of Educational Sciences «AUXILIUM» , Rome, Italy

## Abstract

**Introduction:**

Positive writing (PW) consists in a written treatment of real or imagined events, processed with a positive connotation. The technique has been proven useful and effective for increasing psychological well-being. It derives from the expressive writing (EW) methodology developed by James Pennebaker.

**Objectives:**

The objective of this study is to analyze the effects of positive writing (PW) in a group of healthy subjects. The psychological variables measured following the application of PW are six mood states: tension, depression, anger, vigor, fatigue and confusion. These are preliminary data from work that is still in progress.

**Methods:**

Two groups were randomly formed (one experimental and one control) and wrote for 3 consecutive days on different topics. The experimental group wrote for 20 minutes a day about the most rewarding experience of their life, while the control group described, again for 20 minutes a day, a topic with a low emotional connotation (description of their home). Three administrations (baseline, 3-day follow-up and 10-day follow-up) of the POMS (Profile of Mood States) psychological test were carried out on study participants.

A statistical analysis such as analysis of variance (2-way ANOVA for repeated measures) was used to analyze the effects of positive writing in relation to the different parameters considered, between the groups (Experimental Group vs Control Group) in three different times (baseline, 3 days, 10 days).

**Results:**

Statistically significant decreases were recorded in the experimental group in confusion (Factor C) in the 10-day measurement (7.44 VS 5.00 p < 0.01) and in fatigue (factor S) (5, 94 VS 3.88; p < 0.05).

**Conclusions:**

These data demonstrate how positive writing can lead to beneficial psychological effects. In particular, this study examined the effects of writing about one’s real life experiences and highlighted beneficial psychological/cognitive effects (decreased confusion) and psychophysical (decreased feelings of fatigue). Focusing attention on one’s positive experiences therefore produces improvements on a cognitive level for the sensations that concern clarity and linearity of thought and reduction of feelings of psycho-physical fatigue.

**Disclosure of Interest:**

None Declared

